# Local cytokine transcription in naïve and previously infected sheep and lambs following challenge with *Teladorsagia circumcincta*

**DOI:** 10.1186/1746-6148-10-87

**Published:** 2014-04-09

**Authors:** Nicola M Craig, David W Smith, Judith A Pate, Ivan W Morrison, Pamela A Knight

**Affiliations:** 1 Jarrett Building, Institute of Biodiversity, Animal Health and Comparative Medicine, School of Veterinary Medicine, University of Glasgow, Bearsden Rd, Glasgow G61 1QH, UK; 2 Moredun Research Institute, Midlothian, UK; 3 Division of Pathology, College of Medicine and Veterinary Medicine, The University of Edinburgh, Edinburgh, UK; 4 The Roslin Institute, Midlothian, UK; 5 INTO Glasgow Caledonian University, Glasgow, UK

**Keywords:** Cytokine, Abomasum, *T. Circumcincta*, Sheep

## Abstract

**Background:**

The abomasal helminth *Teladorsagia circumcincta* is one of the most economically important parasites affecting sheep in temperate regions. Infection is particularly detrimental to lambs, in which it can cause pronounced morbidity and severe production losses. Due to the spreading resistance of this parasite to all classes of anthelmintic drugs, teladorsagiosis is having an increasingly severe impact on the sheep industry with significant implications for sheep welfare. Protective immunity develops slowly, wanes rapidly and does not appear to be as effective in young lambs. To investigate the development of immunity to *T. circumcincta* in sheep and lambs, we used cytokine transcript profiling to examine differences in the abomasal mucosa and gastric lymph node of naïve and previously infected sheep and lambs following challenge.

**Results:**

The results of these experiments demonstrated that the abomasal mucosa is a major source of cytokines during abomasal helminth infection. A local Th2-type cytokine response was observed in the abomasal mucosa and gastric lymph node of the previously infected sheep and lambs when compared with those of the naïve during the early stages of infection. In contrast, a pro-inflammatory component more was evident in the abomasal mucosa and gastric lymph node of the naïve sheep when compared with those of the previously infected, which was not observed in the lambs.

**Conclusions:**

The greater levels of Th2-type cytokine transcripts in both the abomasum and gastric lymph node of the previously infected compared with naïve sheep and lambs emphasises the importance of these mechanisms in the immune response to *T. circumcincta* infection. Younger lambs appear to be able to generate similar Th2-type responses in the abomasum suggesting that the increased morbidity and apparent lack of resistance in younger lambs following continuous or repeated exposure to *T. circumcincta* is unlikely to be due to a lack of appropriate Th2-type cytokine production.

## Background

The abomasal nematode *Teladorsagia circumcincta* is one of the most economically important parasites to affect the farming of sheep and goats in temperate and subtropical areas [1-5]. This parasite is especially detrimental to younger lambs, in which it can cause pronounced morbidity and severe production losses if not controlled. Control of teladorsagiosis currently relies upon the use of anthelmintics, and is complicated by the increasing incidence of resistance to these drugs, which has had a significant impact on the sheep industry and implications for sheep welfare [6-11].

Infection with *T. circumcincta* causes functional changes in the abomasum, including a rapid reduction in gastric secretion [12,13] and suppression of gastric acid production, leading to increased abomasal pH [14-17] and reduced activation of pepsinogen. Gross pathology shows areas of inflammation of the abomasal mucosa, in which parasitized glands can be located at the centre of nodular lesions. Histologically, there is epithelial hyperplasia and thickening of the mucosa, which may progress to mucosal sloughing [18], reduction in the prevalence of parietal cells, increased numbers of mucus neck cells [3,17], and infiltration of mast cells, eosinophils and T cells [19,20]. Breakdown of the junctions between epithelial cells, possibly by mast cell proteases [21], causes increased mucosal permeability, loss of protein and fluid into the gut lumen, and allows passage of pepsinogen into the blood [3,22-24]. Clinical consequences of infection range from sub-optimal weight gain, to inappetence, weight loss, protein deficiency and diarrhoea [22,25].

Repeated exposure to *T. circumcincta* eventually results in the development of protective immunity [16,26-28], manifested as rapid expulsion of infective larvae, inhibited parasite development and growth, and reduced fecundity of adults [29-32]. Vaccination has therefore been suggested as a viable alternative to anthelmintic treatment as a means of control [33-37]. However, the immune responses to gastrointestinal helminths take far longer to develop, and develop to a lesser extent, in young lambs [38-40]. Improved understanding of the differences between young lambs and older sheep, in terms of immune responses to gastrointestinal helminths, will aid the development of gastrointestinal helminth vaccines which are effective in young animals.

While the immune response to *T. circumcincta* is similar to that of other gastrointestinal helminths in sheep and cattle, which provoke a predominantly Th2 phenotype response with associated up-regulation of Th2-type cytokines [41-45], the local cytokine profile generated in the abomasum in response to *T. circumcincta* infection in naïve and previously infected animals has not yet been reported.

In young lambs, deficiency in generating effective protective immunity to gastrointestinal parasites has been demonstrated by a number of studies in which lambs under 6 months of age failed to develop immunity following exposure to helminths including *H. contortus* and *Trichostrongylus colubriformis*. In contrast, the same exposure resulted in protective immune responses in older sheep [40,46-50]. However, while apparently deficient in generating protective immunity to helminth infections in comparison to older sheep, young lambs have been shown to produce immune responses to vaccines containing *H. contortus* intestinal antigen; which in one study resulted in a significant reduction in pasture contamination with *H. contortus* larvae [51].

Previous studies of the immune response to *T. circumcincta* have demonstrated that lambs at 4.5 months of age were capable of generating resistance to infection, as shown by increased larval stunting and developmental arrest following later challenge. However, this resistance was measurably less than that generated using the same infection protocol in 10-month-old sheep, in which almost all larvae were arrested early in development [26,31,38].

Cytokine responses in the gastric lymph node of yearling sheep have previously been examined during a trial study of infection with *T. circumcincta*[45]. Here we set out to compare the cytokine responses in both the abomasum and gastric lymph node of naïve and previously infected yearling sheep over the course of a challenge infection with *T. circumcincta*, and to investigate whether these responses differ in a similar experiment using 5-month-old lambs.

## Results

Over the course of a challenge infection with *T. circumcincta*, transcription of interleukins 1β (IL-1β), 2 (IL-2), 4 (IL-4), 5 (IL-5), 6 (IL-6), 10 (IL-10), 12 (IL-12p_40_), 13 (IL-13) and 18 (IL-18), interferon gamma (IFNγ), transforming growth factor beta one (TGFβ_1_) and tumour necrosis factor alpha (TNFα) was examined in the abomasal mucosa and gastric lymph node of naïve and previously infected (PI) yearling sheep and 5-month-old lambs. The experimental design of this study is summarised in Table 1. Due to differences in breed and surgical status, comparisons can only be made between naive and PI animals, and between the day 0 and day 2 yearlings.

**Table 1:** **Design of *T. circumcincta *** **infection experiments**

**Experiment**	**Age**	**Breed**	**Group**	**Trickle infection**^**a**^	**Kill day following challenge**^**b**^				
					**0**	**2**	**5**	**10**	**21**
2	Yearling	Dorset × Suffolk	Naive	-	-	-	6^c^	6^c^	6^c^
			Previously Infected	+	-	-	6^c^	6^c^	-
4	Yearling	Scottish Blackface × Bluefaced Leicester	Naive	-	6	6	-	-	-
			Previously Infected	+	6	6	-	-	-
6	5 months	Dorset × Suffolk	Naive	-	4	-	6^c^	6^c^	6^c^
			Previously Infected	+	4	-	6^c^	6^c^	6^c^

^a^2000 *T. circumcincta* L3 administered three times per week for 8 weeks.

^b^Quoted figure is the number of animals in the group killed on the corresponding day following challenge.

^c^Common gastric lymph duct cannulated 4–7 days prior to challenge infection (day 0).

### Parasitology

Detailed post-mortem parasitology data obtained from the animals used in these experiments, including gastric contents and mucosal digests, have been presented elsewhere [52-54]. Worm counts are listed in Table S1 and S2 (see Additional file 1). As expected, significantly higher numbers of *T. circumcincta* were recovered from the abomasum of naive yearlings than from the corresponding PI group (day 2, P = 0.024; day 5, P = 0.005; day 10 P < 0.001). In contrast the numbers of *T. circumcincta* recovered from the naïve and PI 5-month-old lambs was not significantly different on days 5 and 10, but were significantly higher in the naïve animals on day 21 (P = 0.024).

### Mast cell counts

**Figure 1:**
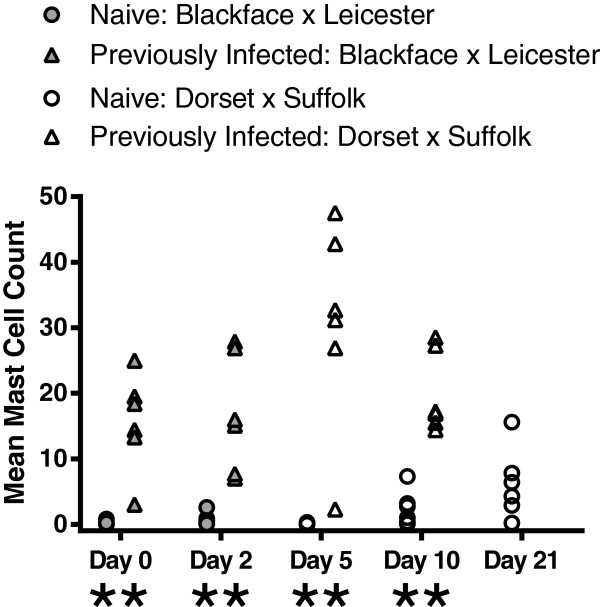
**Abomasal mast cell counts from naïve and previously infected yearling sheep.** Previously infected sheep were orally infected with 2,000 L3 larvae three times per week for eight weeks. All sheep were treated with Levamisole (7.5 mg/kg) then challenged one week later with 50,000 L3 larvae and killed on day 0, 2, 5 or 10 following challenge. An additional group of naïve sheep were killed on day 21 following challenge. Data analysed using Mann–Whitney U-test for non-parametric data with a 95% confidence interval, n = 6 sheep per group for each time point. Significant difference between naïve and previously infected groups, **P < 0.01. Significant difference between day 0 and corresponding day 2, ^#^P < 0.05.

Mast cell numbers in the abomasal mucosa were examined as an indicator of the extent of the Th2 phenotypic response which occurred following infection with *T. circumcincta* in the yearling groups (Figure 1). Throughout the course of the challenge infection PI yearlings were found to have significantly higher numbers of mast cells in the abomasal mucosa than the corresponding naive animals (days 0, 2, 5 and 10, P = 0.0051). Hypertrophy of the abomasal mucosa was also noted following exposure to *T. circumcincta*, and this was marked in the PI animals (data not shown).

### Cytokine transcript levels in the yearling abomasal mucosa

**Figure 2:**
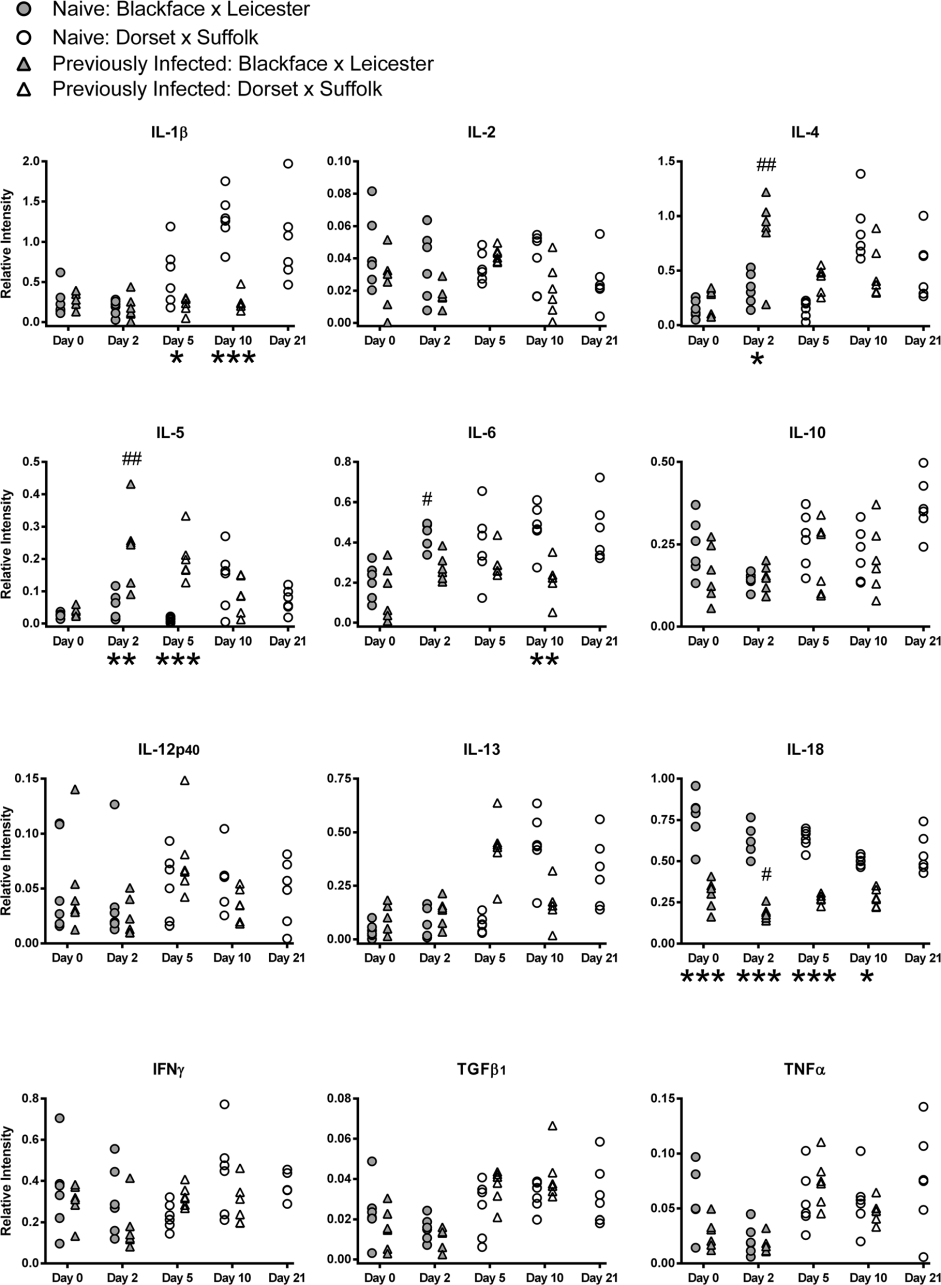
**Cytokine transcript levels in yearling abomasal mucosa.** Relative cytokine transcript levels in samples of abomasal mucosa, in comparison to ATPase, from naïve and previously infected yearling sheep (Scottish Blackface × Bluefaced Leicester and Dorset × Suffolk) following challenge with *T. circumcincta*. Previously infected sheep were orally infected with 2,000 L3 larvae three times per week for eight weeks. All sheep were treated with Levamisole (7.5 mg/kg) then challenged one week later with 50,000 L3 larvae and killed on day 0, 2, 5 or 10 following challenge. An additional group of naïve sheep were killed on day 21 following challenge. Analysed using Tukey’s test with a 95% confidence interval, n = 6 sheep per group for each time point. Significant difference between naïve and previously infected groups: *P < 0.05, **P < 0.01, ***P < 0.001. Significant difference between day 0 and corresponding day 2: ^#^P < 0.05, ^##^P < 0.01.

Cytokine transcript levels relative to ATPase in samples of abomasal mucosa from naïve and previously infected sheep at various time points following challenge with *T. circumcincta* are presented in Figure 2.

A more pronounced Th2-type response was observed in the PI yearlings compared with the naïve. This was demonstrated by a significantly greater abundance of IL-4 (P = 0.0289) and IL-5 (P = 0.0059) transcripts in the PI yearlings than in the naïve group on day 2, which in the case of IL-5 was also found on day 5 (P < 0.0001). This response also took place earlier in the PI animals, as demonstrated by a greater level of IL-4 (P = 0.0012) and IL-5 (P = 0.0081) transcripts on day 2 compared with day 0 in the PI yearlings, but not in the naïve. There was a trend towards a greater abundance of IL-13 transcripts in the PI sheep than the naive on day 5, then in the naive than the PI on day 10, but these differences were not significant.

In contrast to the marked differences in Th2-type cytokine transcript levels, no significant differences were found in the abundance of transcripts of the Th1-type cytokines IL-2, IL-12p_40_ and IFNγ, in the yearling abomasal mucosa samples.

A considerable pro-inflammatory component to the local immune response to *T. circumcincta* was indicated in the naïve yearlings by a greater abundance of IL-1β, IL-6 and IL-18 transcripts when compared with the PI group: IL-1β transcript levels were greater in the naïve yearlings on days 5 (P = 0.0219) and 10 (P = 0.0006); IL-6 transcript levels were greater in the naïve yearlings on day 10 (P = 0.0068); and IL-18 transcript levels were greater in the naïve yearlings throughout the experiment (days 0 and 2, P < 0.0001; day 5, P = 0.0001; day 10, P = 0.0181). Transcript levels of IL-6 were also greater on day 2 in the naïve yearlings compared with day 0 (P = 0.0366). In contrast, the abundance of transcripts of IL-18 was less in the PI yearlings by day 2 compared with day 0 (P = 0.0453). No significant differences were found in levels of TNFα, IL-10 or TGFβ_1_ transcripts in the yearling abomasal mucosa.

### Cytokine transcript levels in the 5-month-old lamb abomasal mucosa

**Figure 3:**
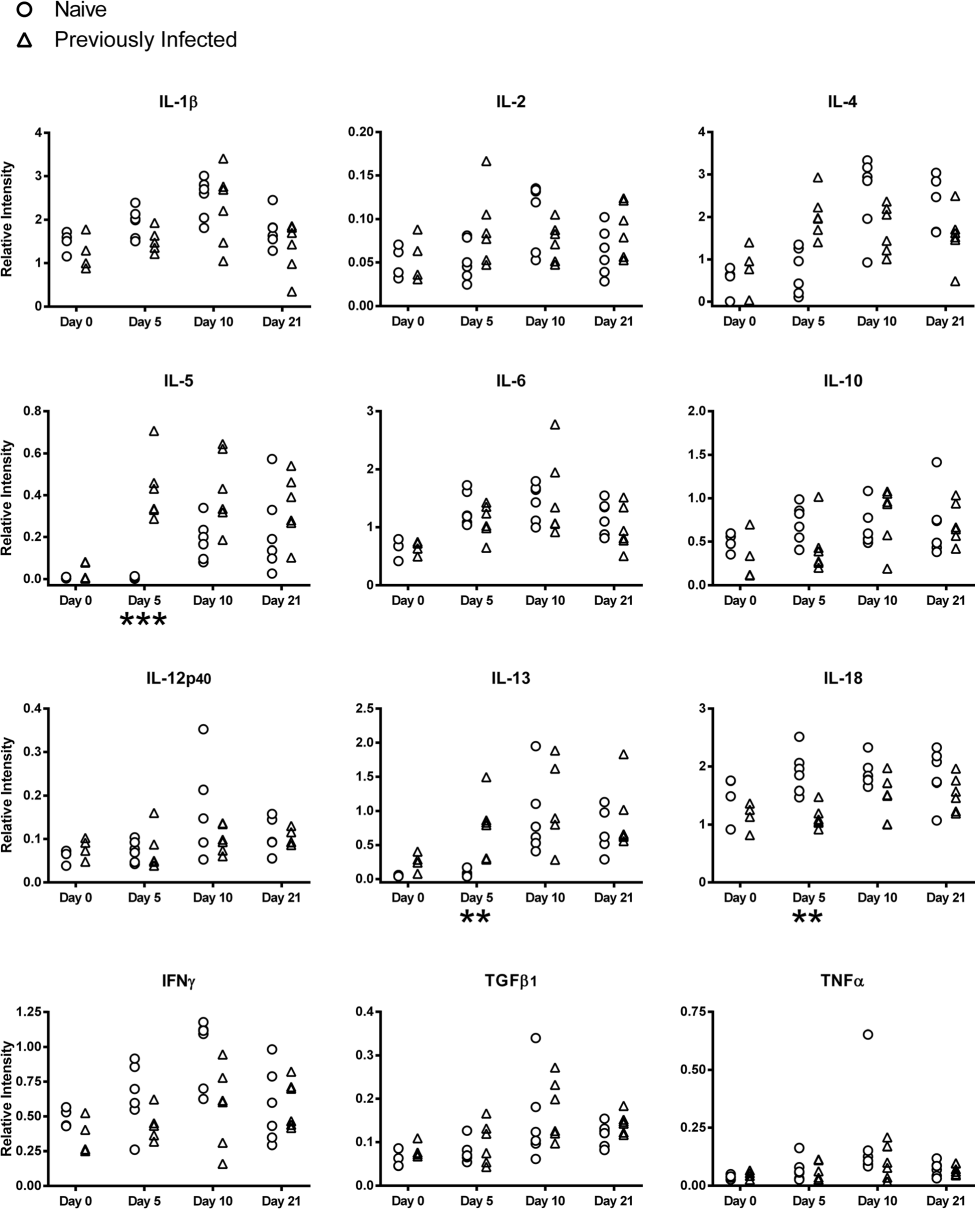
**Cytokine transcript levels in 5-month-old lamb abomasal mucosa.** Relative cytokine transcript levels in samples of abomasal mucosa, in comparison to ATPase, from naïve and previously infected 5-month-old Dorset × Suffolk lambs following challenge with *T. circumcincta*. Previously infected lambs were orally infected with 2,000 L3 larvae three times per week for eight weeks. All lambs were treated with Levamisole (7.5 mg/kg) then challenged one week later with 50,000 L3 larvae and killed on day 0, 5, 10 or 21 following challenge. Analysed using Tukey’s test with a 95% confidence interval, n = 4–6 lambs per group for each time point. Significant difference between naïve and previously infected groups: *P < 0.05, **P < 0.01, ***P < 0.001.

Cytokine transcript levels relative to ATPase in samples of abomasal mucosa from naïve and previously infected 5-month-old lambs at various time points following challenge with *T. circumcincta* are presented in Figure 3.

The 5-month-old lambs demonstrated a Th2-type cytokine response which was similar to the yearling sheep. On day 5 the abundance of both IL-5 (P = 0.0001) and IL-13 (P = 0.0022) transcripts was greater in the PI lambs than the naïve.

Like the yearlings, no significant differences in the level of transcript for the Th1 cytokines IL-2, IL-12p40 or IFNγ were found in the 5-month-old lambs.

A pro-inflammatory component to the immune response was suggested by a greater abundance of IL-18 transcripts in the naïve lambs compared with the PI on day 5 (P = 0.0061), mimicking the pattern observed in the yearlings. No significant differences in the levels of IL-1β or IL-6 transcript levels were found between the naïve and PI 5-month-old lambs.

No significant differences in the abundance of IL-10 or TGFβ_1_ transcripts in the abomasal mucosa were observed between naïve and PI 5-month-old lambs.

### Cytokine transcript levels in the yearling gastric lymph node

**Figure 4:**
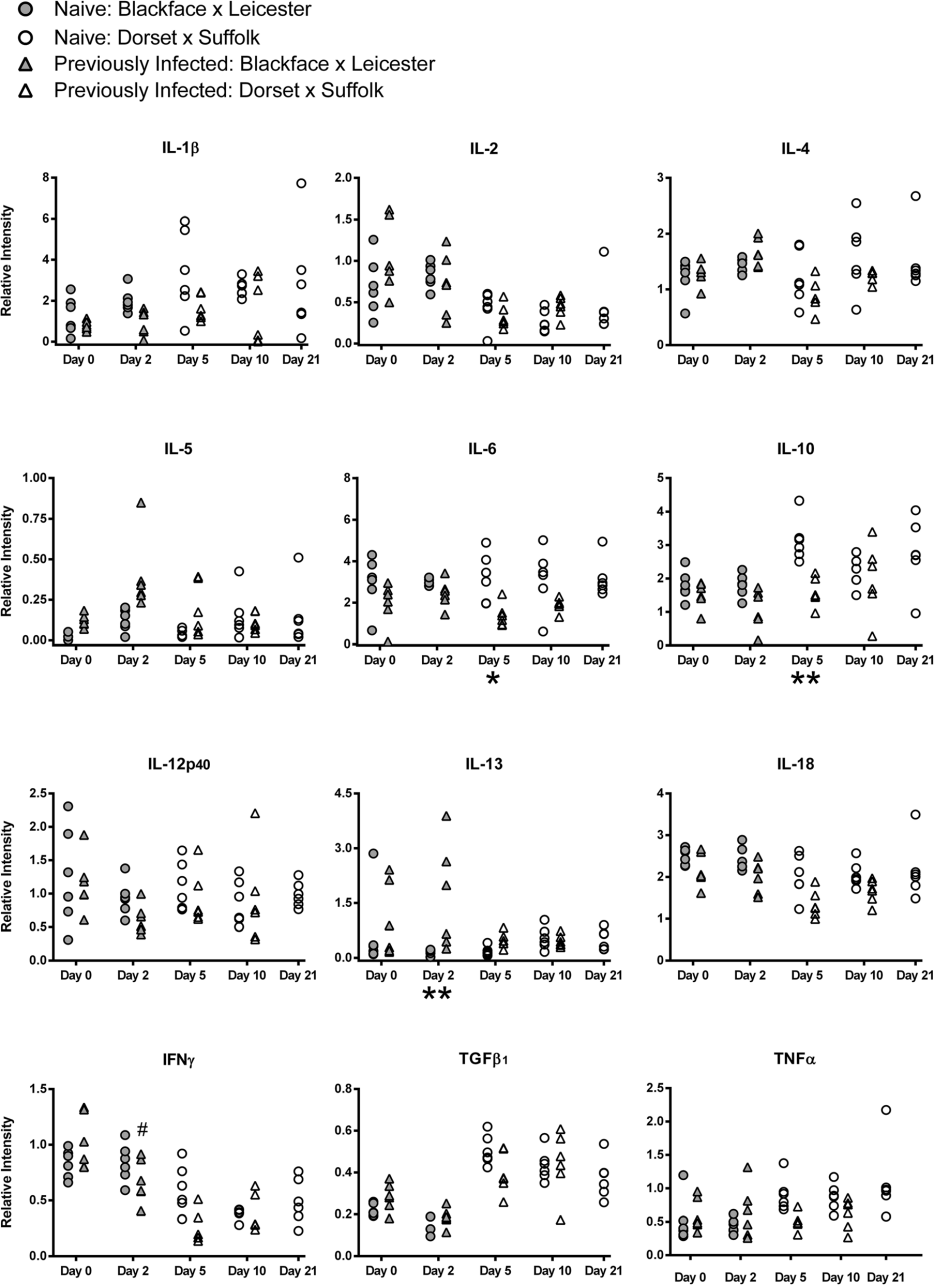
**Cytokine transcript levels in yearling gastric lymph node.** Relative cytokine transcript levels in samples of gastric lymph node, in comparison to ATPase, from naïve and previously infected yearling sheep (Scottish Blackface × Bluefaced Leicester and Dorset × Suffolk) following challenge with *T. circumcincta*. Previously infected sheep were orally infected with 2,000 L3 larvae three times per week for eight weeks. All sheep were treated with Levamisole (7.5 mg/kg) then challenged one week later with 50,000 L3 larvae and killed on day 0, 2, 5 or 10 following challenge. An additional group of naïve sheep were killed on day 21 following challenge. Analysed using Tukey’s test with a 95% confidence interval, n = 6 sheep per group for each time point. Significant difference between naïve and previously infected groups: *P < 0.05, **P < 0.01, ***P < 0.001. Significant difference between day 0 and corresponding day 2: ^#^P < 0.05, ^##^P < 0.01.

Cytokine transcript levels relative to ATPase in samples of gastric lymph node from naïve and previously infected sheep at various time points following challenge with *T. circumcincta* are presented in Figure 4.

Transcript levels of IL-13 were found to be greater in the PI group than the naïve on day 2 (P = 0.0028). However, in contrast to the abomasal mucosa, no significant differences in IL-4 or IL-5 transcript abundance in the gastric lymph node were observed between the naïve and PI yearlings.

As in the abomasal mucosa, no significant differences were found in the abundance of IL-2, IL-12p40 or IFNγ transcripts in the gastric lymph node between naïve and PI yearlings. Interestingly, transcript levels of the Th1 cytokine IFNγ were significantly reduced in the gastric lymph node of the PI group on day 2 (P = 0.0296) compared with day 0.

The abundance of IL-6 transcripts is lesser in the PI group than in the naïve on day 5 (P = 0.0337), however, in contrast with the abomasal mucosa, there were no significant differences in the abundance of IL-1β or IL-18 transcripts in the gastric lymph node between naïve and PI groups. No significant difference in TNFα transcript levels were observed between the naïve and PI yearlings.

The level of IL-10 transcripts in the naïve group on day 5 was found to be significantly greater than that of the corresponding PI group (P = 0.0057). There was no significant difference in the TGFβ_1_ transcript levels in the gastric lymph node between naïve and PI yearlings.

### Cytokine transcript levels in the 5-month-old lamb gastric lymph node

**Figure 5:**
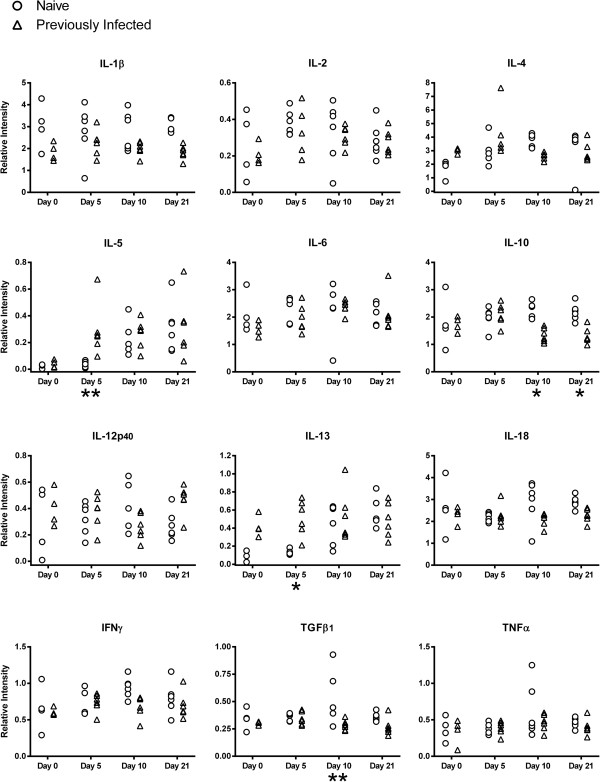
**Cytokine transcript levels in 5-month-old lamb gastric lymph node.** Relative cytokine transcript levels in samples of gastric lymph node, in comparison to ATPase, from naïve and previously infected 5-month-old Dorset × Suffolk lambs following challenge with *T. circumcincta*. Previously infected lambs were orally infected with 2,000 L3 larvae three times per week for eight weeks. All lambs were treated with Levamisole (7.5 mg/kg) then challenged one week later with 50,000 L3 larvae and killed on day 0, 5, 10 or 21 following challenge. Analysed using Tukey’s test with a 95% confidence interval, n = 4–6 lambs per group for each time point. Significant difference between naïve and previously infected groups: *P < 0.05, **P < 0.01, ***P < 0.001.

Cytokine transcript levels relative to ATPase in samples of gastric lymph node from naïve and previously infected 5-month-old lambs at various time points following challenge with *T. circumcincta* are presented in Figure 5.

Similar to those findings in the abomasal mucosa, significantly greater Th2-type cytokine transcript levels were detected in the gastric lymph node of PI 5-month-old lambs compared with naïve. On day 5 the abundance of both IL-5 (P = 0.0025) and IL-13 (P = 0.0202) transcripts was greater in the PI lambs than the naïve. No significant differences between naïve and PI lambs were found in the transcript levels of either the Th1-type cytokines IL-2, IL-12p_40_ and IFNγ, or in the pro-inflammatory cytokines IL-1β, IL-6 and TNFα, in the gastric lymph node.

The abundance of IL-10 transcripts was found to be greater in the naïve than the PI lambs on days 10 (P = 0.0157) and 21 (P = 0.0207), and that of TGFβ_1_ transcripts was found to be greater in the naïve than the PI lambs on days 10 (P = 0.0052).

## Discussion

The fact that significantly fewer *T. circumcincta* were recovered from PI yearlings than from the naive throughout these experiments indicates that more effective anti-parasite responses were elicited in these animals following trickle immunisation compared with the naïve. In contrast, no significant difference in the numbers of recovered worms was found in the 5-month-old animals until day 21, indicating that the any anti-parasite responses generated in the lambs during trickle infection were less effective, or had waned within the seven day period following clearance of the trickle infection. The significantly greater numbers of mast cells observed in the abomasal mucosa of the previously infected yearlings compared with the naïve animals on day 0 is consistent with a Th2-type phenotypic response during the eight week trickle infection of these sheep. In a separate investigation of the immune responses taking place in the gastric lymph of these animals, lower post-mortem worm counts and a greater degree of larval stunting were associated with a stronger IgA response and an earlier blast cell response in the efferent lymph of the previously infected yearlings than in the naïve challenged animals [53,54].

The observed difference in levels of transcripts for IL-4 and IL-5 in the abomasal mucosa following infection of yearlings with *T. circumcincta* is consistent with studies of *H. contortus* infection of sheep [44] and *O. ostertagi* infection of cattle [42]. During the early stages of the immune response these cytokines were transcribed to a greater extent in the previously infected than the naïve sheep following challenge, which is also consistent with the responses observed during *H. contortus* infection [44]. In contrast to observations of *O. ostertagi* infection in cattle [42], transcript levels of IFNγ were not significantly greater in the abomasum of yearling sheep infected with *T. circumcincta*.

These results indicate that cells within the abomasal mucosa itself appear to be a major source of cytokine production, and show evidence of a marked Th2-type immune response occurring during infection of yearling sheep with *T. circumcincta*. The importance of Th2-type responses following infection with *T. circumcincta* is emphasised by observations from other studies using tissue from the same sheep, showing that Th2-induced molecules such as sheep intelectin 2 [55-57], calcium activated chloride channel-1 [58], ovine galectin-14 and sheep mast cell protease-1, were increased following challenge [55,56,59]. Sheep intelectin 2 was also produced earlier in the previously infected sheep [55-57]. Concurrent inflammation was indicated in the abomasal mucosa of the naïve challenged animals by greater levels of IL-1β and IL-6 transcripts. This pro-inflammatory influence is consistent with the mucosal inflammation which is a feature of *T. circumcincta* infection.

The timing of the cytokine responses in the yearling abomasum is interesting. The early up-regulation of IL-4 and IL-5 in the abomasal mucosa of the previously infected sheep corresponds with increased anti-parasite responses to *T. circumcincta* in this group. Expulsion of *T. circumcincta* larvae occurred by day 2 in the previously infected group, resulting in significantly lower worm burdens compared with the naïve challenged sheep [53,54].

In the yearling gastric lymph node there are few significant differences between the naïve and PI animals. The greater abundance of IL-13 transcripts in the previously infected yearling gastric lymph node again indicates a Th2-type cytokine response. Transcripts of IL-2 and IFNγ appear to be less abundant in the later stages of infection, which is consistent with the Th2-type response and concurrent suppression of Th1-type cytokine levels observed in the gastric lymph node during *O. ostertagi* infection of cattle [42].

The differing cytokine responses in the gastric lymph node compared with the abomasal mucosa illustrates the differing roles in the immune response allocated to each tissue. Early stage cytokine signalling in the abomasal mucosa is likely to be important in the initial focussing of the immune response both in terms of helminth-specific responses and their location in the abomasal mucosa, for example IL-4 may play a role in stimulating dendritic cells to induce appropriate gut-associated homing receptor expression in the T cells activated in the lymph node [60]. The lymph node, as the site of induction of the adaptive immune response by basophils and dendritic cells arriving from the mucosa via the afferent lymph, is predominantly engaged in the activation, polarisation and tissue-specific targeting of lymphocytes prior to recirculation [61,62]. Recirculating lymphocytes homing to the mucosa [62,63] are therefore able to direct and amplify later stage cytokine responses in the abomasum. The cytokine responses observed in the abomasal mucosa of 5-month-old lambs were broadly similar to those found in yearling sheep, suggesting a Th2-type response which occurred earlier and more prominently in the previously infected lambs, and an inflammatory response which was more pronounced in naïve challenged animals. However, in contrast to the yearlings, these lambs also demonstrated a Th2-type response in the gastric lymph node with no suppression of Th1-type cytokine transcript levels.

This indicates that the lower resistance and increased morbidity associated with helminth infection in young lambs is unlikely to be due to an inability to produce local Th2 or inflammatory responses. This is consistent with the findings of previous studies of *H. contortus* infection of 3-month-old lambs, which demonstrated a clear Th2-type response indicated by recruitment of mast cells and eosinophils, and increased transcription of IL-4, IL-5 and IL-13 in the abomasal mucosa, but demonstrated no significant suppression of IL-12 or IFNγ in the gastric lymph node [44]. It is possible that the influence of Th1-type cytokines in the lymph node may be inhibiting anti-parasitic immune responses due to suppression of Th2-type cytokine production and/or antagonism of their effects in the abomasum in younger lambs.

The more prominent transcript levels of IL-10 in the gastric lymph node of naïve 5-month-old lambs following infection with *T. circumcincta*, when compared with previously infected lambs, is consistent with the findings in yearling sheep. However, this discrepancy in IL-10 transcript abundance was found earlier in the yearling sheep. It is possible that a delay or reduction in the development of regulatory responses in the younger lambs may contribute to the increased morbidity due to helminth infection in these animals.

The importance of the interplay between Th1, Th2, pro-inflammatory and regulatory cytokines is illustrated by recent studies by Hassan and colleagues examining the immune responses to *T. circumcincta* infection in 12-week-old lambs carrying the DRB1*1101 allele of the major histocompatibility complex DRB1 [64]. This allele is associated with resistance to *T. circumcincta* and, as well as an earlier reduction in worm burdens, carrier lambs demonstrated an earlier shift in abomasal cytokine transcription from Th1 to regulatory and Th2 cytokines compared with more susceptible non-carrier lambs.

## Conclusions

The data presented here indicate that the abomasal mucosa is a major source of cytokine signalling during the development of a local Th2-phenotype response and the generation of protective immunity to *T. circumcincta* in yearling sheep.

The more pronounced Th2-type cytokine transcription in the abomasal mucosa of the previously infected compared with naïve yearling sheep emphasises the importance of these mechanisms in the immune response to *T. circumcincta* infection and the development of resistance to infection.

Younger lambs appear to be able to generate similar Th2-type responses in the abomasum to those of yearlings. However, it is possible that a lack of Th1-type cytokine suppression in the gastric lymph node may be a factor in the apparent lack of efficacy of these responses in generating resistance in younger lambs following continuous or repeated exposure to *T. circumcincta*.

## Methods

### Animals and experimental design

All animal experiments described in this study were carried out in accordance with Moredun Research Institute, Roslin Institute and R (D) SVS guidelines, subject to approval by the Moredun Research Institute’s Animal Experiments and Ethics Committee, and authorised under the UK Animals (Scientific Procedures) Act 1986.

A series of experiments were set up to compare the immune responses of yearling sheep and 5-month-old lambs which had been previously infected with an eight-week trickle infection of *T. circumcincta* L3 larvae, with those of helminth-naïve animals following a challenge dose of 50,000 larvae. Naïve and previously infected sheep and lambs were killed at several time-points following challenge (Table 1). All animals were reared indoors under conditions designed to prevent accidental infection with helminths.

Each experiment contained one group of animals which was exposed to a trickle infection of 2000 *T. circumcincta* L3 larvae administered by oral gavage three times per week for eight weeks. These previously infected (PI) animals were then treated with Levamisole (7.5 mg/kg) before being given a single challenge dose of 50,000 *T. circumcincta* L3 larvae seven days later (day 0). The remaining helminth-naïve animals in each experiment did not receive a trickle infection, but were otherwise treated identically to the PI group, including Levamisole treatment. Sheep were killed by captive-bolt stunning and exsanguination. At post-mortem samples of mucosa were scraped off the underlying muscle in the fundic region of the abomasum. Gastric lymph node samples were also collected, and both tissues were preserved in RNAlater (Applied Biosystems, Warrington, UK) and stored at −20°C until use. Separate samples of abomasal mucosa from the yearling groups were also fixed using 4% paraformaldehyde.

Sheep and lambs killed on days 5, 10 and 21 underwent cannulation of the common gastric lymph duct 4–7 days prior to challenge in order to collect lymph samples for use in concurrent studies [52-54]. It was known from previous experiments using this model that cellular and humoral immune responses to *T. circumcincta* infection all occurred by day 9 in previously infected yearling sheep [26], therefore the PI yearling group was not extended to day 21.

The *T. circumcincta* L3 larvae used in each experiment were from the same batch of an anthelmintic susceptible *T. circumcincta* isolate, which had been stored at 4°C for up to one month prior to use.

### RNA extraction

RNA was extracted from the RNAlater preserved abomasal mucosa and gastric lymph node samples as described previously [45] using a Stratech Beadbeater-8 (Stratech Scientific, Soham, UK) and 1 mm^3^ zirconia-silica beads (Thistle Scientific, Glasgow, UK), RNeasy Mini Kit (Qiagen, Crawley, UK) with β-mercaptoethanol (Thermo Fisher Scientific Inc., Waltham, USA), Qiashredder columns (Qiagen, Crawley, UK) and Qiagen RNAse-free DNAse (Qiagen, Crawley, UK). Complete removal of native DNA contamination required additional DNAse treatment in solution (DNA-free kit, Applied Biosystems, Warrington, UK). The 260 nm absorbance of a 1:5 dilution of sample RNA was measured using a Cecil CE2021 2000 series spectrophotometer (Cecil Instruments, Cambridge, UK) and used to calculate the RNA concentration. RNA purity was assessed by determining the ratio 260/280 nm absorbance.

### Reverse transcription of mRNA into cDNA

Reverse transcription of 1 μg total RNA was set up on ice using a Reverse Transcription Kit (Promega, Southampton, UK) and a Techgene thermocycler (Techne, New Jersey, USA) as previously described [45]. RNA controls were set up by diluting 0.5 μg of sample RNA in 50 μl of RNAse-free water. These control samples were used to check for native DNA contamination during PCR. Both cDNA and RNA controls were stored at -20°C until use.

### PCR amplification of ovine cytokine cDNA

The oligonucleotide primers and PCR cycling conditions used to detect cDNA specific for ovine IL-1β, IL-2, IL-4, IL-5, IL-6, IL-10, IL-12p_40_, IL-13, IL-18, TGFβ_1_, TNFα, IFNγ and ATPase have been previously published [45,65]. ATPase, a constitutively expressed ‘housekeeping’ gene, was used both as a control to assess the quality of the RT product and as a reference for quantification of cytokine mRNA. Primer specificity was checked using PCR product sequencing as described previously [45].

PCR reactions were set up on ice using Taq DNA polymerase (Roche Diagnostics GmbH, Mannheim, Germany) and 250 ng cDNA as described previously [45]. Mastermix only controls to which no cDNA was added were used to check for DNA contamination of the PCR reaction mixture. The reactions were heated to 94°C for 2 min for the initial denaturation, then cycled through 40 seconds at 94°C, annealing at an appropriate temperature for 40 seconds and extension at 72°C for 2 minutes. Thermocycling was concluded by a final extension of 5 minutes at 72°C. Techne Gradient (Techne, New Jersey, USA) and PerkinElmer GeneAmp PCR System 2400 (PerkinElmer, Boston, USA) thermocyclers were used.

A 10 μl aliquot of each PCR product was migrated in 1.3% TBE-agarose gel containing 0.01% GelRed (Biotium, Inc., Hayward, USA). 1Kb Plus DNA ladder was included as a molecular weight marker and gels were photographed using a Bio-Rad Molecular Imager FX (Bio-Rad Laboratories Ltd., Hemel Hempstead, UK). Kodak 1D Analysis software was then used to determine the density of the PCR product bands on each image. To normalise for variation in cDNA levels during PCR, the level of cytokine mRNA transcript was compared relative to the level of ATPase mRNA transcript for each sample; the values were presented as the ratio of the cytokine PCR product band density over that of the corresponding ATPase PCR product.

### Abomasal mucosal mast cell counts

Paraformaldehyde-fixed samples of the abomasal mucosa from the yearling sheep were trimmed, embedded in paraffin and longitudinally sectioned. Sections were de-waxed in ethanol, then washed and stained for 30 minutes using chloroacetate esterase staining solution consisting 0.1 M potassium phosphate (pH 6.0), 40 mg Fast Garnet GBC salt, 5 mg Naphthol AS-D Chloroacetate, 1 ml DMSO in a total volume of 81 ml (Thermo Fisher Scientific Inc., Waltham, USA) [66-68].

Stained sections were washed using distilled water and mounted using polyvinylpyrrolidone, then examined using a Leitz Laborlux S microscope (Leica Microsystems GmbH, Wetzlar, Germany). The number of mucosal mast cells were counted using a 1 mm^2^ eyepiece graticule, as closely as possible orientated with the top edge along the epithelial surface, at ten locations along the section under **×** 250 magnification. The mean mast cell count for each sample was recorded.

### Statistical analysis

Statistical analyses were performed using Minitab Statistical Software release 15 for Windows (Minitab Inc., Pennsylvania, USA). Data were checked for deviation from a normal distribution using an Anderson-Darling test. Where data for a particular factor was found to deviate from normality it was transformed to fit a normal distribution using a Johnson transformation selected using a p-value of 0.05. The data was then analysed using a general linear model technique considering immune status (naive or PI), and kill day nested within immune status. Where a significant difference was indicated Tukey’s tests were used post-hoc to compare the means of naive and PI groups on the same day, and the mean of the yearling day 2 group to that of the corresponding day 0. Significance was indicated by a p-value <0.05 and confidence intervals used were 95%.

Abomasal mast cell counts from the yearlings were not normally distributed and could not be successfully transformed. Kruskal-Wallis test, followed by Mann–Whitney U-tests, was therefore used to look for differences between pairs of groups. Again significance was indicated by a p-value <0.05 and confidence intervals used were 95%.

## Competing interests

The authors declare that they have no competing interests.

## Authors’ contributions

NMC planned and carried out the main body of the work (molecular analyses, mast cell counts and statistical analysis), interpreted the results and drafted the manuscript. WDS conceived and supervised the experimental infections. JAP assisted with post-mortem sample collection, processing and histology. WIM conceived and obtained the central VTRI funding (grant number VT0102). PAK conceived and supervised this project and helped to draft the manuscript. All authors read and approved the final manuscript.

## Supplementary Material

Additional file 1: Table S1 Lists yearling worm counts and Table S2 lists 5-month-old lamb worm counts. Click here for file
