# Supplementary material for: Local cytokine transcription in naïve and previously infected sheep and lambs following challenge with Teladorsagia circumcincta
Source: BMC Vet Res. 2014 Apr 9;10:87. doi: 10.1186/1746-6148-10-87 (PMC4234407; doi:10.1186/1746-6148-10-87)
Supplement: Additional file 1: Table S1 — Lists yearling worm counts and Table S2 lists 5-month-old lamb worm counts. [file 1746-6148-10-87-S1.pdf]

Table S1 – Yearling worm counts.

Naive	Kill day following challenge	Worm count	Percentage of L4	Previously Infected	Kill day following challenge	Worm count	Percentage of L4
	0	-	-		0	-	-
		-	-			-	-
		-	-			-	-
		-	-			-	-
		-	-			-	-
		-	-			-	-
	2	3478	-		2	2618	-
		12305	-			1324	-
		8285	-			3085	-
		15429	-			578	-
		3200	-			1252	-
		7242	-			4629	-
	5	5818	0.0		5	4273	23.4
		6073	2.4			451	8.1
		8591	0.0			198	55.0
		7220	1.4			40	0.0
		2821	0.0			0	0.0
		2923	2.9			652	0.0
	10	9871	3.4		10	36	0.0
		8471	5.8			1707	94.7
		11564	0.4			6642	11.1
		15836	0.0			19	0.0
		11853	2.4			2967	77.8
		17355	2.2			5185	45.9
	21	11564	0.0				
		3780	0.0				
		3863	0.0				
		16647	0.2				
		2791	0.0				
		11855	0.3				

Table S2 – 5-month-old lamb worm counts.

Naive	Kill day following challenge	Worm count	Percentage of L4	Previously Infected	Kill day following challenge	Worm count	Percentage of L4
	0	-	-		0	-	-
		-	-			-	-
		-	-			-	-
		-	-			-	-
	2	-	-		2	-	-
		-	-			-	-
		-	-			-	-
		-	-			-	-
		-	-			-	-
		-	-			-	-
	5	5600	-		5	1160	-
		4160	-			5440	-
		8080	-			4760	-
		4440	-			1040	-
		4360	-			4200	-
		10880	-			3520	-
	10	21416	0.0		10	14542	12.0
		16309	0.0			5580	3.7
		1554	45.5			954	11.5
		12820	2.0			1719	43.0
		17694	0.0			11010	6.0
		22140	0.0			8551	5.3
	21	13880	-		21	80	-
		13560	-			2720	-
		640	-			3440	-
		18880	-			720	-
		33600	-			11720	-
		21440	-			2800	-
